# Safety and efficacy of fully-covered self-expandable metal stent placement for refractory stomal stenosis

**DOI:** 10.1055/a-2777-8929

**Published:** 2026-01-27

**Authors:** Ahamed A. Khalyfa, Navkiran K. Randhawa, Rahil Desai, Mahnoor Inam, Varshita Goduguchinta, Kamran Ayub

**Affiliations:** 1 21710 Gastroenterology, University of Iowa Health Care, Iowa City, United States; 2 1421 Gastroenterology, Augusta University, Augusta, United States; 3 6203 IM, Franciscan Health Olympia Fields, Olympia Fields, United States; 4 Gastroenterology, Southwest Gastroenterology, New Lenox, United States; 5 6203 Franciscan Health Olympia Fields, Olympia Fields, United States; 6 21431 Gastroenterology, Silver Cross Hospital, New Lenox, United States

**Keywords:** Endoscopy Upper GI Tract, Malignant strictures, Endoscopy Lower GI Tract, Colorectal cancer, Inflammatory bowel disease, Stenting

## Abstract

**Background and study aims:**

Stomas, critical in managing various gastrointestinal conditions, can lead to complications like stomal stenosis, affecting 2% to 15% of patients and causing significant morbidity. Traditional treatments such as balloon dilation and surgical revisions often fail to provide lasting relief. This study investigated a novel, minimally invasive approach using fully-covered self-expanding metal stents (FCSEMSs) to address refractory stomal stenosis effectively.

**Patients and methods:**

Five patients with recurrent stomal strictures unresponsive to conventional treatments underwent stent placement. Etiologies included Crohn’s disease, ischemia, and post-surgical fibrosis. Stents were selected based on stricture characteristics, ranging from 10 to 16 mm in diameter and 6 to 10 cm in length. Technical success was defined as successful deployment without immediate complication; clinical success was defined as sustained symptom resolution during follow-up. Outcome measures included abdominal girth, pain reduction (visual analog scale), vomiting, and dietary tolerance. Follow-up periods ranged from 8 weeks to 6 months.

**Results:**

All procedures were technically successful. Mean abdominal girth decreased from 114 ± 4.2 cm (range 108–119) to 103 ± 3.7 cm (range 99–108). Pain scores decreased from a mean of 8 ± 1.1 to 2 ± 1.2. Vomiting, bloating, and dietary tolerance improved in most patients. No stent migration or erosions/ulcer was observed during follow-up. Four patients had follow-up to 6 months without restenosis. One patient had restenosis with successful re-stenting within a 6-month follow-up period.

**Conclusions:**

FCSEMSs represent a safe and effective minimally invasive alternative for refractory stomal stenosis, with promising short-term outcomes.

## Introduction

Stomas of the digestive tract play an essential role in managing many gastrointestinal conditions, and their creation significantly impacts patient outcomes, with over 130,000 stomas established annually in the United States 1 . Common stoma types include Hartmann’s end colostomy, loop colostomy, and ileostomy. These are often required for conditions such as colorectal cancer, inflammatory bowel disease, diverticular disease with obstruction, and penetrating bowel injuries 2 .

Stomal stenosis affects 2% to 15% of patients, causing discomfort and necessitating additional surgical interventions 3 . Common causes include ischemia, skin scarring, inflammatory diseases, and residual neoplasms. Although mild stenosis can often be managed with serial balloon dilation, severe stenosis frequently requires surgical revision. However, both options carry significant limitations 4 5 .

This study explored the efficacy and safety of fully-covered self-expanding metal stents (FCEMSs) in treating refractory stomal stenosis, offering a potentially effective alternative to invasive surgical procedures.

## Patients and methods

**Fig. 1:**
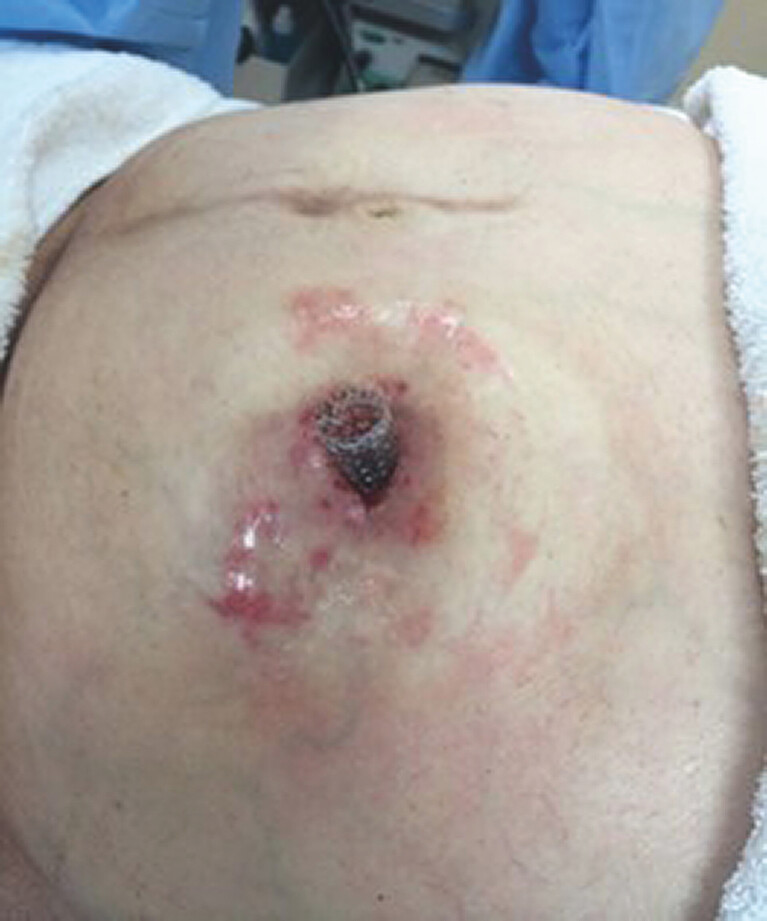
Fully-covered SEMS in place across stomal stricture (skin-level stoma).

Five patients with refractory stomal strictures and bowel obstruction were treated with SEMSs ( Fig. 1 ). All had failed previous interventions, defined as persistence or recurrence of obstructive symptoms within 4 weeks following balloon dilation or surgical revision. Etiologies included Crohn’s disease (2 cases) and colorectal cancer (3 cases).

Stent placement was performed in the endoscopy suite under monitored anesthesia care with fluoroscopic guidance. Stents were selected based on anatomical location, degree of stenosis, and provider experience. Two patients received Alimaxx 14 mm × 10 cm (braided, fully covered), two patients with Crohn’s disease received GORE VIABIL 10 mm × 6 cm (laser-cut, fully covered), and one received Alimaxx 16 mm × 10 cm.

Initial access was obtained using a 0.035-inch wire and 6-mm balloon dilation. Stents were secured externally using silk sutures. Intended dwell time was 4 weeks, but actual retention ranged from 4 to 8 weeks due to scheduling logistics. All stents were removed electively.

Outcome measures included abdominal girth, pain scores (visual analog scale), vomiting, and dietary tolerance (liquids and solids). Clinical success was defined as resolution of obstructive symptoms within 2 weeks and maintenance during follow-up.

## Results

All stents were placed successfully without immediate complication. Mean abdominal girth decreased from 114 ± 4.2 cm (range: 108–119) to 103 ± 3.7 cm (range: 99–108). Pain scores improved from 8 ± 1.1 to 2 ± 1.2. Three patients had vomiting prior to stenting; all resolved post-treatment. All five reported pre-procedure bloating, which resolved in every case ( Table 1 ).

**Table:** **Table 1** Demographic and clinical characteristics

Patient	Age	Gender	Diagnosis	Stent type	Duration of stent	Prior interventions	Abdominal girth pre/post	Pain pre/post	Vomiting	Bloating
1	67	M	Colorectal cancer	Alimaxx 14 mm	7 weeks	Surgery	115/104	7/2	Yes→No	Yes→No
2	75	M	Colorectal cancer	Alimaxx 14 mm	8 weeks	Balloon	114/105	9/2	Yes→No	Yes→No
3	52	F	Crohn’s disease	GORE VIABIL 10 mm	6 weeks	Balloon	112/102	8/3	No	Yes→No
4	38	F	Crohn’s disease	GORE VIABIL 10 mm	6 weeks	Surgery	108/99	8/1	No	Yes→No
5	63	M	Colorectal cancer	Alimaxx 16 mm	4 weeks	Balloon	119/108	8/2	Yes→No	Yes→No

Dietary tolerance improved substantially. One of five patients could tolerate liquids before treatment; all five tolerated liquids post-treatment. Solid food tolerance improved from 0/5 to 4/5. Four patients were followed for 6 months without recurrence ( Table 2 ). One patient had recurrence with subsequent re-stenting.

**Table:** **Table 2** Grouped outcome summary before and after treatment.

Parameter	Before Treatment	After Treatment
Abdominal girth (cm)	114 ± 4.2 (108–119)	103 ± 3.7 (99–108)
Pain score (VAS)	8 ± 1.1 (7–9)	2 ± 1.2 (1–3)
Tolerating liquid diet	1/5	5/5
Tolerating solid diet	0/5	4/5
Vomiting	3/5	0/5
Bloating	5/5	0/5
VAS, visual analog scale.		

## Discussion

Stomal stenosis remains a challenging complication in 2% to 15% of patients with stomas 1 2 . Existing treatments such as balloon dilation and surgical revision have notable limitations. Balloon dilation often provides only temporary relief, requiring repeated interventions and causing discomfort to patients 3 4 . Surgical revision, although effective in some cases, is invasive, costly, and associated with higher morbidity, particularly in patients with a history of multiple surgeries or compromised health 5 6 .

This study demonstrates that FCEMSs provide a minimally invasive alternative that addresses many of these limitations. The minimally invasive nature of this procedure is particularly valuable in patients with prior surgical history or comorbidities. No stent migration or erosion was observed, likely due to external fixation and use of anti-migration flared ends. Symptom improvement was consistent across patients, with significant reductions in abdominal girth and pain, complete resolution of vomiting and bloating, and improved oral intake. These outcomes meet our definition of clinical success. Technical success was achieved in all cases.

Compared with existing data on balloon dilation, which is associated with recurrence rates up to 60% 5 , and surgical revision, which carries complication rates up to 20% 8 9 , our cohort experienced minimal adverse events during follow-up. Despite these promising results, the study’s small sample size limits generalizability of the findings. Future research should focus on larger, multicenter trials to confirm these outcomes and explore long-term efficacy and durability 10 11 . In addition, studies comparing this technique directly with surgical revision and other noninvasive methods could further establish its place in the treatment algorithm for stomal stenosis [11].

## Conclusions

FCSEMSs are a safe and effective treatment for refractory stomal stenosis, offering significant improvements in quality of life while reducing the need for surgery. These findings support their use as an alternative to more invasive interventions in high-risk patients.
